# Analysis of copy number variations in the sheep genome using 50K SNP BeadChip array

**DOI:** 10.1186/1471-2164-14-229

**Published:** 2013-04-08

**Authors:** Jiasen Liu, Li Zhang, Lingyang Xu, Hangxing Ren, Jian Lu, Xiaoning Zhang, Shifang Zhang, Xinlei Zhou, Caihong Wei, Fuping Zhao, Lixin Du

**Affiliations:** 1 National Center for Molecular Genetics and Breeding of Animal, Institute of Animal Sciences, Chinese Academy of Agricultural Sciences, Beijing 100193, People’s Republic of China; 2 Institute of Animal Science, Inner Mongolia Academy of Agricultural & Animal Husbandry Sciences, Hohhot, Inner Mongolia Autonomous Region, 010031, People’s Republic of China; 3 Chongqing Academy of Animal Sciences, Chongqing, 402460, People’s Republic of China

## Abstract

**Background:**

In recent years, genome-wide association studies have successfully uncovered single-nucleotide polymorphisms (SNPs) associated with complex traits such as diseases and quantitative phenotypes. These variations account for a small proportion of heritability. With the development of high throughput techniques, abundant submicroscopic structural variations have been found in organisms, of which the main variations are copy number variations (CNVs). Therefore, CNVs are increasingly recognized as an important and abundant source of genetic variation and phenotypic diversity.

**Results:**

Analyses of CNVs in the genomes of three sheep breeds were performed using the Ovine SNP50 BeadChip array. A total of 238 CNV regions (CNVRs) were identified, including 219 losses, 13 gains, and six with both events (losses and gains), which cover 60.35 Mb of the sheep genomic sequence and correspond to 2.27% of the autosomal genome sequence. The length of the CNVRs on autosomes range from 13.66 kb to 1.30 Mb with a mean size of 253.57 kb, and 75 CNVRs events had a frequency > 3%. Among these CNVRs, 47 CNVRs identified by the PennCNV overlapped with the CNVpartition. Functional analysis indicated that most genes in the CNVRs were significantly enriched for involvement in the environmental response. Furthermore, 10 CNVRs were selected for validation and 6 CNVRs were further experimentally confirmed by qPCR. In addition, there were 57 CNVRs overlapped in our new dataset and other published ruminant CNV studies.

**Conclusions:**

In this study, we firstly constructed a sheep CNV map based on the Ovine SNP50 array. Our results demonstrated the differences of two detection tools and integration of multiple algorithms can enhance the detection of sheep genomic structure variations. Furthermore, our findings would be of help for understanding the sheep genome and provide preliminary foundation for carrying out the CNVs association studies with economically important phenotypes of sheep in the future.

## Background

In recent years, genome-wide association studies (GWAS) have successfully uncovered single-nucleotide polymorphisms (SNPs) associated with complex diseases or traits [1]. With the rapid development of chip array-based genotyping techniques, thousands of genomic submicroscopic structural variations have been found in the human genome [2]. As a main genetic form of submicroscopic structural variation copy number variations (CNVs) are widely distributed in the human genome and influence gene expression, phenotypic variation and adaptation by disrupting genes and altering gene dosage [2-5]. Numerous studies showed that CNVs contributed to both disease susceptibility and phenotypic diversity [2,5]. Now, CNV is increasingly considered to be an important and abundant source of genetic variation and phenotypic diversity [5,6].

Investigations on CNVs have been successively carried out in human and other species [7-13]. In the domestic animals, there are involving in cattle [14-20], dog [21], chicken [22], pig [23,24], goat [25], sheep [26] and rabbit [27]. As for sheep, Fontanesi et al. [26] provided a first comparative map of CNVs of the sheep genome referred to the cattle genome using a cross-species array comparative genome hybridization(aCGH). However, the cross-species analysis based on heterologous hybridization couldn’t identify all detectable CNVRs due to low homology between cattle probes and sheep DNA for some regions and doesn’t show the CNVR distributions on the sheep genome. In addition to CGH, another major platform commonly used to identify CNVs is the SNP array. In SNP array, intensity values of SNPs derived from each sample are used to detect CNVs in each individual. Comparison with two panels, CGH array has excellent performance in signal-to noise ratios, while the SNP array based approach is more convenient for high-throughput analyses and follow-up association studies [28]. With the development of high density SNP arrays, higher resolution of genomic regions can be achieved [29]. Moreover, due to their low cost and high-density, commercial SNP arrays have been widely used for CNV detection in domestic animals, and CNV mapping and functional studies have made important progress. However, there are no reports on CNV detection of sheep based on SNP array data.

In this study, we will investigate genome-wide CNV in three sheep populations. To pursue convincing results, we firstly employ the PennCNV program to analyze Ovine SNP50 genotyping data, and then use other algorithm program, cnvPartition, to validate CNVRs detected by PennCNV. To our knowledge, we will construct the first sheep CNV map based on SNP array data. This research will provide useful addition to the sheep CNV maps, and will provide potential genetic markers for further investigation on the roles of CNV in sheep productive traits and evolutionary adaptation.

## Results

### SNP genotyping

The genomic DNA of 329 individual samples from three sheep breeds (German Mutton sheep, Dorper and Sunite sheep) were genotyped using Illumina OvineSNP50 Genotyping BeadChip according to the manufacturer’s protocol, and the PennCNV (http://www.openbioinformatics.org/penncnv) software was used to identify the CNVs in the sheep genome (Table 1). According to the results of PennCNV, we defined the CNV call filtering criteria to exclude samples with low quality of signal intensity data. After applying the CNV quality control criteria detailed in the “Methods” section, 256 samples (157 German Mutton, 35 Dorper and 64 Sunite sheep) remained for further CNV analyses.

**Table 1:** Population size information in sheep copy number variation analysis

**Breed**	**No, of sheep**^**a**^	**PennCNV**^**b**^	**CNVpartition**^**c**^
German Mutton Sheep	161	157	110
Sunite Sheep	69	64	43
Dorper Sheep	99	35	26
Total	329	256	179

a: total samples before quality control by PennCNV and CNVpartition.

b: The samples that passed the quality control of PennCNV in 329 individuals from three sheep breeds.

c: The samples that passed the quality control of CNVpartition in 329 individuals from three sheep breeds.

### Genome-wide surveys of sheep CNVs

**Figure 1:**
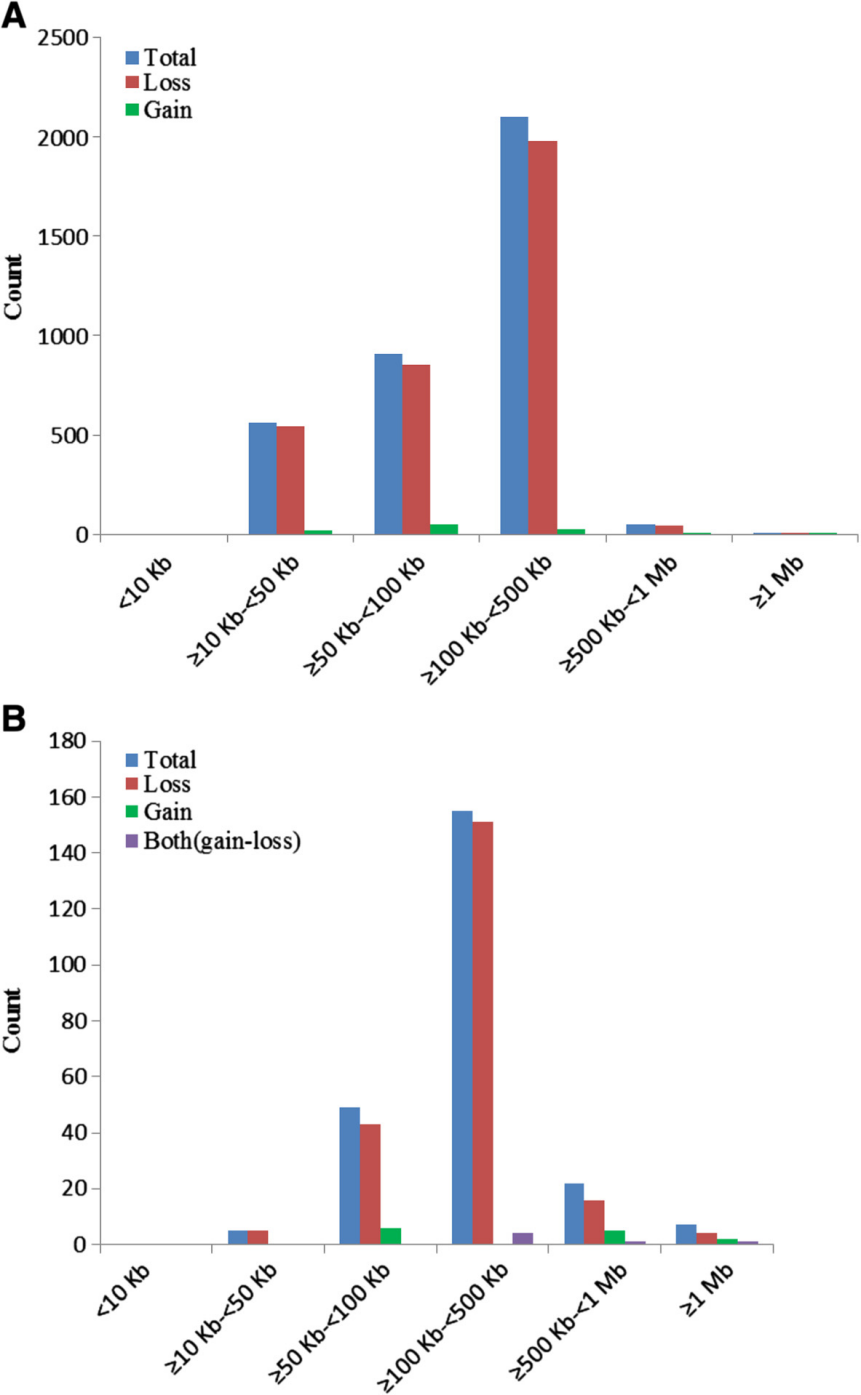
**The size distribution of CNV. **(**A**) The distribution of CNVs size in the three sheep breeds. (**B**) The distribution of CNVRs size in the three sheep breeds. Smaller CNVs (<10 kb) were not identified in this study.

After filtering unreliable CNV calls, we discovered a total of 3624 CNV events (3416 losses and 208 gains), with an average number of 14.16 CNV events per individual. The average and median sizes of CNVs were 144.6 kb and 122.9 kb, respectively (Table 2). We found that approximately 58% of the CNVs ranged from 100 to 500 kb, and 25% ranged from 50 to 100 kb in size distribution (Figure 1A and Additional file 1: Table S1). Smaller CNVs (< 10 kb) were not identified in this study.

**Table 2:** Genomic characteristics of copy number variations in sheep (n = 256)

	**Total number**	**Total length(Mb)**	**Average size (kb)**	**Median size (kb)**	**Loss**	**Gain**	**Both (Gain-Loss)**	**No. of common CNVs (freq ≥ 5 %))**	**No. of common CNVs (5% > freq ≥ 3%))**	**Unique**
Individual CNV	3624	524.11	144.62	122.9	3416	208				
CNVR	238	60.35	253.57	186.92	219	13	6	62	13	72

### Characteristics of CNVRs identified in sheep

**Figure 2:**
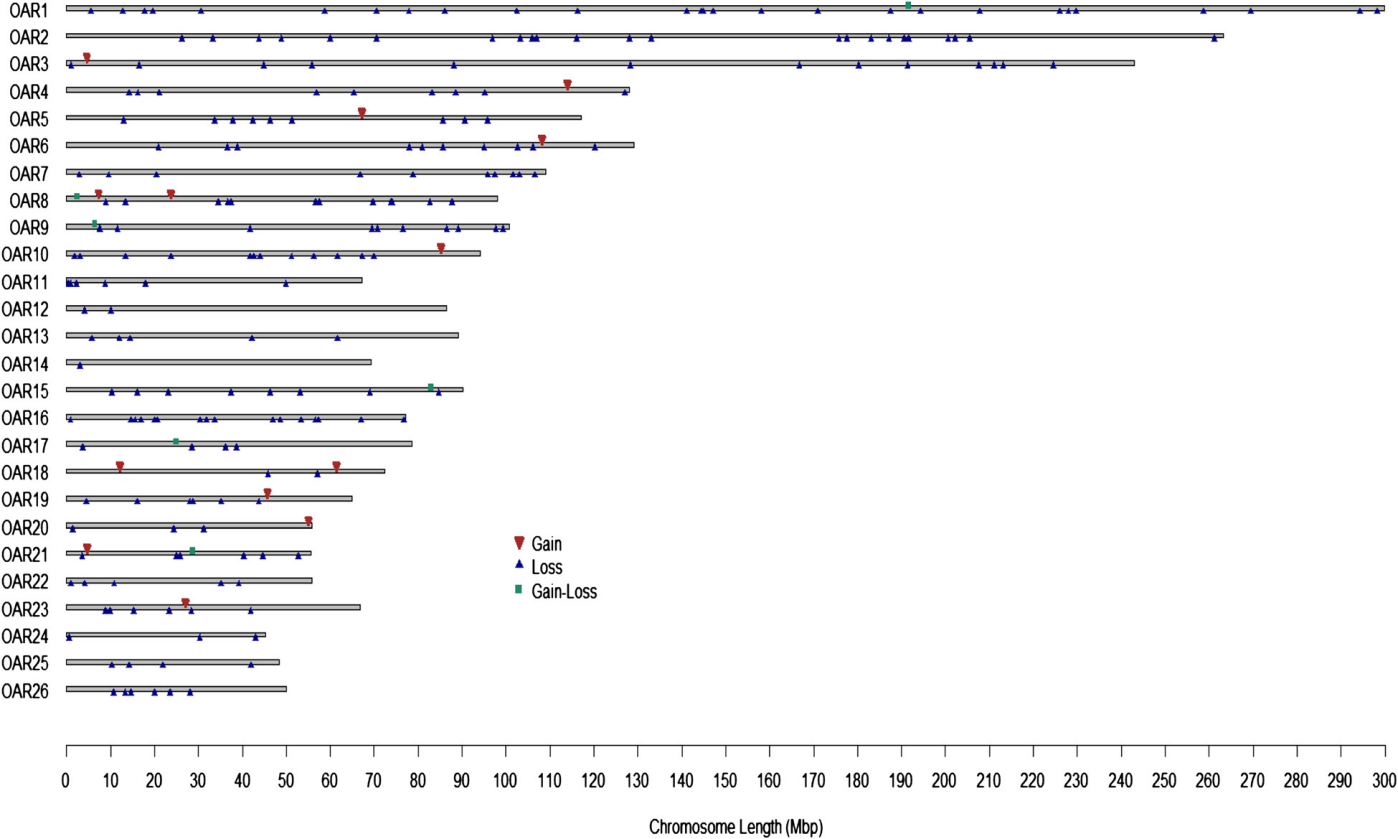
**Map of sheep CNVRs. **Red, blue and green represented the Gain, Loss and both (Gain-Loss), respectively. A total of 238 CNV regions, including 219 Loss events, 13 Gain events, 6 both events(Gain-Loss); 60.35 Mb, ~2.27% of sheep autosomal genome.

CNV regions (CNVRs) were determined by merging the overlapping CNVs identified in all samples, as reported previously [4]. A total of 238 autosomal CNVRs were identified, covering 60.35 Mb of the sheep genomic sequence and corresponding to 2.27% of the autosomal genome sequence (60.35 Mb/2655.6 Mb) and 2.17% of the whole sheep genome (60.35 Mb/2784.7 Mb). More information on CNVRs is also presented in Figure 2 and Additional file 1: Table S3.

These 238 CNVRs, ranging from 13.66 Kb to 1.30 Mb with mean and median sizes of 253.57 and 186.92 kb, respectively (Table 2), included 219 losses, 13 gains, and six with both events (gains and losses). Loss events were present approximately 16.8-fold more than gain events, similar to previously reports of other species [14,16,25,26]. The majority (85%) of CNVRs ranged from 50 kb to 500 kb (Figure 1B and Additional file 1: Table S2). Furthermore, of the 238 CNVRs, 72 CNVRs were found in only one animal (Unique), whereas 166 CNVRs were found in ≥ two animals and breeds (multiple) [20] and 13 CNVRs had a frequency > 3%, 62 CNVRs had a frequency > 5%. (Table 2 and Additional file 2: Table S4). Specifically, the CNVR Ovis aries chromosome (OAR) 9:11627386–12923703 had the highest frequency (65%). In the report by McCarroll *et al.*[30], the copy number polymorphism (CNP) term was coined when describing 75 CNVRs as common CNVRs. In addition, as shown in Table 3, the numbers of CNVRs identified by PennCNV were large difference among three sheep breeds. In the German Mutton, we identified 172 CNVRs, which comprised 72.27% of the total CNVRs detected. In Sunite and Dorper breeds, 134 and 126 CNVRs were detected, corresponding to 56.30% and 52.9% of the total number, while 72 unique CNVRs, 29, 24 and 19 CNVRs were detected in German Mutton, Sunite and Dorper breeds, respectively.

**Table 3:** The CNVR number detected by PennCNV in three sheep breeds

**Breed**	**CNVRs number**^**a**^	**Unique CNVRs**^**b**^
German Mutton Sheep	172	29
Sunite Sheep	134	24
Dorper Sheep	126	19
Total	238	72

a: CNVRs number means the total number detected in one breed.

b: Unique CNVRs means CNVRs only detected in one breed.

### Construction of copy number variation map

We constructed the map of CNVR distribution on the chromosomes based on the sheep whole genome SNP genotyping chips (Figure 2). The results indicated that the CNVs among sheep breeds were non-randomly distributed among the different chromosomes, and that the percentage of CNVRs in the chromosomes varies from 0.46–5.08% (Figure 2 and Additional file 1: Table S3), with the top three highest percentages of CNVRs located on chromosomes 16, 26 and 9 (5.08%, 4.94%, and 4.11%, respectively). This might be due to bias derived by the analysis of the Ovine SNP50 BeadChip. Chromosome 1 had the greatest number and largest length of CNVRs, whereas chromosome 14 had the smallest length and number of CNVRs. The most enriched chromosomes for CNVRs in sheep were chromosomes 1, 2 and 5.

To verify the CNVs detected by PennCNV, we also used the CNVpartition program implemented in Illumina GenomeStudio to analyze the data and detect CNVs. After applying the quality filtering criteria, we identified 179 individuals with CNVs, and 41 CNVRs were determined by merging overlapping CNVs identified across all samples. It should be noted that all 179 individuals were encompassed by 256 individuals passed the quality filtering criteria of PennCNV. The results of CNVRs distributed on chromosomes were similar to those of PennCNV (Additional file 1: Table S3), and the OAR16 showed the greatest enrichment for CNV (18.45%), which was consistent with the result of PennCNV program. But, chromosomes with more five CNVRs were OAR3 and OAR7. The different results might be due to the different algorithms between PennCNV and CNVpartition. In a comparison of the results from the PennCNV and CNVpartition programs, we found 47 CNVRs identified by the PennCNV program (19.7%) that overlapped with the CNVpartition data, and the ‘losses’ and ‘gains’ of 47 CNVRs were consistent with the CNVpartition data. Some CNVRs identified by the CNVpartition analysis were larger in size, resulting in overlapping of multiple CNVRs identified by PennCNV, thus, we segmented these large CNVRs in the CNVpartition results. In total, we obtained 52 CNVRs by CNVpartition analysis (see Additional file 3: Figure S1).

### CNV validation by quantitative PCR

We performed quantitative real-time PCR experiments to evaluate the accuracy of the copy number assignments. Ten putative CNVRs were selected for CNV validation, these ten CNVRs represent different predicted status of copy numbers (i.e., gain and loss) and CNVR frequencies varied from 0.30 to 15.20%, of which four contained known sheep RefSeq genes in the UCSC database (see Additional file 4: Table S5); the remaining six CNVRs were selected randomly. We performed 60 qPCR assays in 49 animals, out of the 60 qPCR assays, 16 were in agreement with prediction by PennCNV. When counting the CNVRs, 6 out of the 10 CNVRs confirmed the existence of copy number variations in at least one qPCR assay, whereas primer pairs in four other regions did not work (Additional file 4: Table S5).

### Functional enrichment analysis of CNVRs

We found five sheep RefSeq genes partially or entirely encompassing four CNVRs. To further analyze the gene content of the 238 CNVRs, we used a BLASTN search for homologous human and cattle sequences using the UCSC table browser tool. There were 1043 RefSeq homologous human genes located within or partially overlapping with 127 CNVRs of the 238 CNVRs and similarly, there were 270 RefSeq homologous cattle genes located within or partially overlapping with 106 CNVRs of the 238 CNVRs (Additional file 5: Table S6). To assess the functional annotation of these CNVRs, we conducted gene ontology analysis (GO) using the Database for Annotation, Visualization and Integrated Discovery (DAVID) functional annotation tool. We retrieved 1313 gene symbols (homologous genes) to load into the DAVID tool. We selected ‘human’ as the background, and the 563 corresponding human gene IDs were identified in DAVID. GO analysis indicated that the genes overrepresented in DAVID were involved in olfactory receptor activity, sensory perception of smell, sensory perception of chemical stimulus, sensory perception, cognition, neurological system processes, G-protein coupled receptor protein signaling pathway, cell surface receptor linked signal transduction, plasma membrane as well as integral and intrinsic membrane components, protein polymerization and microtubule-based movement. In addition to the above gene functions, the DAVID pathway results showed genes involved in olfactory transduction and pathogenic *Escherichia coli* (Table 4). We also found that a number of CNVRs overlapped with known disease-related genes in the DGV database. A total of 341 disease-related genes reported in DGV were located either wholly or partially within CNVRs in our results (see the Additional file 6: Table S7).

**Table 4:** Functional enrichment analysis of CNVRs

**Molecular function**	**GO Name**	**Count**	**%**	***P *value**	**FDR**
GO:0004984	olfactory receptor activity	178	32.07207	5.862E-158	8.27E-155
GO:0034212	peptide N-acetyltransferase activity	3	0.540541	0.006224705	8.432901467
GO:0004596	peptide alpha-N-acetyltransferase activity	3	0.540541	0.006224705	8.432901467
GO:0005344	oxygen transporter activity	4	0.720721	0.007951513	10.65236765
GO:0005218	intracellular ligand-gated calcium channel activity	3	0.540541	0.01014878	13.40426119
GO:0005219	ryanodine-sensitive calcium-release channel activity	3	0.540541	0.01014878	13.40426119
GO:0042043	neurexin binding	3	0.540541	0.020398884	25.23210706
GO:0004683	calmodulin-dependent protein kinase activity	4	0.720721	0.026734392	31.7727638
Biological Process	GO Name	Count	%	*P* value	FDR
GO:0007608	sensory perception of smell	179	32.25225	1.284E-157	2.15E-154
GO:0007606	sensory perception of chemical stimulus	179	32.25225	6.6567E-148	1.11E-144
GO:0007600	sensory perception	181	32.61261	4.4745E-105	7.48E-102
GO:0050890	cognition	185	33.33333	3.8912E-100	6.51E-97
GO:0007186	G-protein coupled receptor protein signaling pathway	189	34.05405	2.47502E-87	4.14E-84
GO:0050877	neurological system process	193	34.77477	3.0364E-85	5.08E-82
GO:0007166	cell surface receptor linked signal transduction	201	36.21622	5.69264E-59	9.52E-56
GO:0051258	protein polymerization	9	1.621622	0.000194423	0.324648125
GO:0007018	microtubule-based movement	11	1.981982	0.00446377	7.208705512
GO:0015671	oxygen transport	4	0.720721	0.008202995	12.8682872
GO:0051225	spindle assembly	4	0.720721	0.017671563	25.78281061
GO:0015669	gas transport	4	0.720721	0.020691633	29.50783311
GO:0007158	neuron adhesion	3	0.540541	0.020837821	29.68360693
GO:0035176	social behavior	4	0.720721	0.023976546	33.35959701
GO:0044065	regulation of respiratory system process	3	0.540541	0.034182249	44.10238982
GO:0002087	regulation of respiratory gaseous exchange by neurological system process	3	0.540541	0.034182249	44.10238982
GO:0007625	grooming behavior	3	0.540541	0.049984805	57.57958749
Cellular Component	GO Name	Count	%	*P* value	FDR
GO:0005886	plasma membrane	241	43.42342	5.86933E-35	7.69E-32
GO:0016021	integral to membrane	289	52.07207	1.23979E-32	1.62E-29
GO:0031224	intrinsic to membrane	293	52.79279	1.36024E-31	1.78E-28
GO:0005833	hemoglobin complex	4	0.720721	0.005887046	7.444488585
Pathway	Pathway name	Count	%	*P* value	FDR
hsa04740	Olfactory transduction	170	30.63063	2.80E-137	3.05E-134
hsa05130	Pathogenic Escherichia coli infection	8	1.441441	0.027376	25.8675045
OMIM_DISEASE		Count	%	*P* value	FDR
Genetic correlates of brain aging on MRI and cognitive test measures: a genome-wide association and linkage analysis in the Framingham Study		3	0.540541	0.011938	12.52341992
Genome-wide association and linkage analyses of hemostatic factors and hematological phenotypes in the Framingham Heart Study		3	0.540541	0.026364	25.7439374
C1r/C1s deficiency, combined		2	0.36036	0.034033	32.00636058

Abbreviation: FDR, false discovery rate (Benjamini and Hochberg method).

### Comparison with other ruminant CNV studies

To compare CNVRs identified by SNP platform in sheep genome which were overlapped with CNVRs reported in other ruminants, we migrated 238 CNVRs from ISGC Ovis aries 1.0 to Btau_4.0 using UCSC liftOver tool [31] and 230 CNVRs were obtained on Btau_4.0 assembly. And then we compared these CNVRs with those previous CNV studies (Additional file 7: Table S8), including four experiments that two were carried out in sheep, goat and two in cattle using aCGH platform [15,20,25,26] and other two in cattle using the Illumina BovineSNP50 BeadChip [14,16]. Comparing sheep CNVRs detected by us and Fontanesi et al. [26], we found three CNVRs overlapped. In addition, there were found two sheep CNVRs matched goat CNVRs identified with aCGH panel [25]. Comparing sheep CNVRs with four other cattle CNVRs reported by Liu et al. [20]. Fadista et al. [15]. Hou et al. [16] and Bea et al. [14], we obtained 54 of 230 CNVRs mapped on Btau_4.0 assembly overlapped with cattle CNVRs with aCGH panel or SNP panel.

## Discussion

In recent years, CNVs have been increasingly recognized as an important source of genetic variation and phenotypic diversity [4,5,30]. A number of CNVs associated with diseases have been found in human genetic researches, involving autoimmune disorders, schizophrenia, autism, as well as infectious and cardiovascular diseases [32,33]. In addition, CNVs have been shown to play a role in pharmacokinetics in terms of drug efficacy and toxicity [34]. Therefore, the systematic analysis and characterization of CNVs improves our understanding of genetic variation and is an important tool for deciphering the role of CNV in the heritability of complex traits. In the past few years, with the development of high-density genotyping arrays, detection of CNVs using SNP genotyping arrays has become a cost-effective and efficient approach. Fine-scale CNV maps for human and other species have been constructed and refined [18,35,36].

In this study, we used whole genome genotyping based on Ovine SNP50 BeadChip arrays to identify CNVs in the sheep genome. After genotyping 329 samples using the Illumina platform from three sheep breeds, the signal intensity (LRR) and allelic intensity (BAF) ratio of all samples were exported using the Illumina GenomeStudio software (Illumina Inc., San Diego, CA, USA). In order to obtain a higher accuracy, quality control and CNV detection optimization was conducted using the PennCNV software when generating CNV calls [16]. To identify reliable CNV data, other studies have used different criteria for filtering unreliable CNV calls. Jakobsson *et al*. restricted analyses to CNVs with a minimum of 10 markers per CNV to minimize the number of false positives [37]. In our study using PennCNV analysis, we tested the impact of genomic waves on CNV calls and the filtering parameters with or without the –gcmodel option set. Compared with the results from the PennCNV using the –gcmodel option, more CNVs were identified without the –gcmodel option (5418 CNVs) than with the –gcmodel option (5008 CNVs; data not shown). This result was similar to that reported by Hou *et al.*[16]. These discordant calls were likely due to false positives called from the differentiating signal intensities caused by ‘genomic waves’. This further demonstrated that genomic waves have a significant effect on CNV analysis. We also used the ‘filter_cnv.pl’ program to perform sample-level quality control (see Materials and Methods). After this step, 73 further samples were excluded because their intensity data failed to conform to the criteria. Finally, the remaining 256 samples were used for CNV detection. This study focused only on the CNVs in 26 autosomes and excluded chrX and chrUn from our analysis because the chrX sequence and annotations are still not well-known [38], furthermore, their sequences and SNPs were uncertain, including the SNP mapping.

We identified a total of 238 CNVRs in three sheep breeds using the PennCNV detection algorithm. Of these, 219 CNVRs were losses, higher than the proportion of gains (Figure 2). This result might be due to the fact that losses are easier to detect than gains, because the exponential intensity data is linearly correlated with the copy number [39]. To exclude the potential false positive signals, we used another CNV detection software, CNVpartition, which detected 52 CNVRs. Forty-seven regions were consistently identified by both CNV detection software packages. Compared to CNVpartition based on other algorithms, such as QuantiSNP [40], Birdsuite [41], and GADA [42], PennCNV combines multiple sources of information, including the total signal intensity and allelic intensity ratios at each SNP marker to generate a hidden state for copy neutral loss of heterozygosity, the distance between neighboring SNPs, and the allele frequency of SNPs. PennCNV also integrates a computational approach by fitting regression models with GC content to overcome ‘genomic waves’ [43].

We compared our CNV length dataset with another sheep CNV study that was based on a cross species cattle-sheep aCGH experiment using a tiling oligonucleotide array with approximately 385,000 probes designed using the bovine genome. Because our dataset excluded CNV calls in the chrX and chrUn, only autosomal CNVR calls (238 CNVRs) were compared to the autosomal CNV calls of that study (135 CNVRs). As in other CNV studies using aCGH experiments [15,20,25,26], the number of loss events in our dataset was larger than the number of gain events. However, further validation is required as it is also possible that purifying selection occurred [5]. The CNV sizes in our study ranged from 100 to 500 kb (average, 144.6 kb), which differed from those in the work of Fontanesi *et al.* (average, 73.9 kb) [26]. This CNV size difference was likely due to sampling differences or to resolution and genome coverage differences between the two techniques. The SNP genotyping resolution was 50.9 kb (mean probe spacing), whereas that of the aCGH platform was 6.3 kb. A SNP array lacked non-polymorphic probes designed specifically for CNV identification. Thus, only the large CNVRs were identified with the Ovine SNP50 assay. In future experiments, high-density SNP arrays combined with improved CNV calling algorithms could remedy these differences. In addition, we investigated the distribution pattern of these CNVRs in 26 autosomes and determined whether the CNV region was common in three sheep breeds. Interestingly, 75 of the 238 CNVRs had frequency rates of > 3%, whereas 121 of the 238 CNVRs had a frequency rate of < 1% in 256 individuals. This result might have been influenced by the innate limitation of Ovine SNP 50 arrays, and therefore, many common CNVs could have been missed.

We used UCSC gene annotation (http://genome.ucsc.edu) to identify genes that were located within or partially overlapped with CNVRs. Initially, we used a database of known sheep genes to annotate the gene content of CNVRs. However, the annotation results showed only four CNVRs overlapping with five known RefSeq genes in sheep CNVRs (see Additional file 5: Table S6). These results are likely attributable to the fact that the sheep genome is not well-annotated compared to the human genome or that of other domesticated animals [44-48]. The current sheep genome version (ISGC Ovis aries 3.1) is incomplete and known related genes are rarely identified in the sheep RefSeq gene database [38]. Therefore, we performed a BLASTN search for homologous human and cattle sequences using the UCSC table browser tool, and identified 563 genes homologous to human IDs identified in DAVID. We conducted a GO analysis to determine the biological effects of the 563 copy number variant genes (homologous to human genes). Similar to other results for GO analyses, the enriched genes were related to those involved in olfactory receptor (OR) activity, sensory perception of smell, sensory perception of chemical stimulus, sensory perception, cognition, G-protein coupled receptor protein signaling pathway, neurological system processes, cell surface receptor-linked signal transduction, plasma membrane and integral membrane components. Table 4 showed that the genes involved in environmental responses were over-represented in the CNVRs. In this study, functional analysis results were similar to those previously reported in CNV studies in human and other mammals [11,15,19,26,49]. This significant (*p* < 3.51 × 10^-158^) enrichment for OR activity might represent the frequent occurrence of CNVs in gene clusters for OR [50], as previously observed in cattle [19]. In addition, we observed 563 human orthologous genes in sheep CNVRs, of which 341 genes were previously reported in the DGV database (Additional file 6: Table S7). Moreover, we identified CNV regions that may overlap with Online Mendelian Inheritance in Man (OMIM) genes influencing disease susceptibility (Table 4). Notwithstanding, in the great majority of cases, these CNVs encompass genes and thereby directly affect gene dosage by loss or gain in the level of gene expression [2,3,51]. However, some other reports have suggested that CNVs are located preferably in gene-poor regions [52,53] or in noncoding regions, and some studies provide evidence that the CNVs are considered nonpathogenic. Recent studies have described genome-wide distribution of CNVs in regions that encompass noncoding sequences, thereby affecting the regulation of distant target genes [54-57]. This suggested impact of CNVs in noncoding regions requires further elucidation in future studies. As discussed above, functional analysis studies indicated that the CNV genes possess a wide spectrum of molecular functions. However, since the sheep genome is less well-defined, Sheep CNVRs warrant further investigation for their roles in complex traits.

To verify CNV obtained by PennCNV, we conducted quantitative PCR (qPCR) on ten selected CNVRs and compared them with a sample control region known to have no CNVs (the DGAT1 gene fragment). We found that 26.7% of our qPCR assays (16 confirmed/60 total) agree with our CNVRs predictions in these regions. In total, six regions of these CNVRs were validated in at least one qPCR assay. However, it should be noted that another four CNVRs were not confirmed by qPCR (Additional file 4: Table S5). Reasons for this discrepancy between the CNVR analysis of Ovine SNP50 BeadChip data and the qPCR experiment may be due to the fact that the low probe density of the Ovine SNP50 BeadChip made it difficult to identify the true boundaries of CNVRs, resulting in an overestimation of their actual sizes. In addition, the primer pairs might have been designed outside the copy number polymorphic region.

In this research, only a small proportion of CNVRs identified overlapped with other studies. A similar situation was also encountered in human and other mammal CNV studies [10,16,58]. This indicated that a vast amount of CNVs existing had not been detected in the Ovine genome. Furthermore, a comparative analysis of CNVRs identified in sheep, goat and cattle could reveal the evolutionary mechanisms determining CNV formation during the mammalian evolution.

## Conclusions

We firstly constructed a sheep CNV map based on the Ovine SNP50 array. 238 CNVRs were totally identified in the sheep autosomal genome, these CNVRs were non-randomly distributed on chromosomes, and 70% of which (166/238 CNVRs) were shared in > two animals. We also confirmed 6 CNVRs in total of 10 selected CNVRs by qPCR method. Compared with the results of CNV studies based on aCGH, the currently available genome wide SNP assays can infer Ovine CNV efficiently. However, it should be noticed that smaller size CNVs (< 10 kb) were not identified using this SNP panel and only 16 out of the 60 qPCR assays confirmed in this study was likely to be a overestimation of the true number and true boundary of CNVRs in the sheep genome. On the other hand, in this study, we using two detection programs: PennCNV and CNVpartition, to identify the sheep genome CNVs, results indicated that 47 CNVRs identified by the PennCNV (19.7%) overlapped with the CNVpartition data (90.4%), which highlighted the differences and commonalities of the two detection methods. Follow-up studies for high-resolution CNV mapping require improved SNP assay and next-generation sequencing to improve the accuracy of CNV calling. In addition, integration of different algorithms can enhance the detection of genomic structure variations. Furthermore, our findings would be of help for understanding the ovine genome and provide preliminary foundation for carrying out the CNVs association studies with economically important phenotypes of sheep in the future.

## Methods

The experiments were performed on trait records and DNA samples that had been collected previously from animals born in 2010. Because no new animals were handled in these experiments, there are no needs of Animal Care and Use Committee approval for this study.

### Samples and genotyping

Blood samples were collected from 329 six-month-old lambs from three breeds including 161 German Mutton sheep (71 males, 90 females), 99 Dorper (49 males, 50 females), and 69 Sunite sheep (57 males, 12 females) using the TIANamp Blood DNA Kit (Tiangen Biotech Co. Ltd. Beijing, China). Whole Genomic DNAs were genotyped using the Illumina Ovine SNP50 BeadChip that contained 54,241 single nucleotide polymorphisms (SNPs) and the average spacing was 50.9 kb. Two individuals were excluded because of call rate less than 98%. The DNA from the remaining 327 individuals that passed the sample quality control criteria were entered into the subsequent CNV detection and analysis procedures.

### CNV identification and filter quality control

All signal intensity (log R ratio: LRR) and allelic intensity (B allele frequency: BAF) ratios of samples were reported using Illumina GenomeStudio1.0 software for each SNP. The PFB file was calculated based on the BAF of each marker in these populations. The sheep GC model file was generated by calculating the GC content of the 1 Mb genomic region surrounding each marker (500 Kb each side). CNVs were inferred using a Hidden Markov Model (PennCNV, http://www.openbioinformatics.org/penncnv/) [39]. PennCNV quality filters were applied after CNV detection. We used high quality samples with a standard deviation (SD) of LRR < 0.30 and with the default set: BAF drift as 0.01 and waviness factor value between -0.05 and 0.05, respectively. Appropriate LRR adjustments based on the GC model were incorporated in PennCNV. In addition, we used the program argument: the “lastchr 26” in the “detect” argument for specific CNVRs. CNV CNVRs were determined by aggregating overlapping CNVs identified in different animals, as reported previously [2,4]. After filtering the samples, the CNVRs identified by PennCNV were shown (Additional file 2: Table S4). For construction of the CNVR map, we classified the status of these CNVR into three categories, ‘Loss’ (CNVR containing deletion), ‘Gain’ (CNVR containing duplication) and ‘Both’ (CNVR containing both deletion and duplications). In addition,we employed the LiftOver tool to map sheep CNVRs coordinates on the Btau_4.0 version so as to compare the CNVRs detected in sheep genome by SNP array with CNVRs in ruminants.

### CNV calling using CNVpartition

In order to verify the CNVs detected by PennCNV, we employed the CNVpartition software [59] to analyze the same data set as well, with the confidence score threshold of 35. The CNVRs detected were cross-validated by CNVpartition and PennCNV. Furthermore, we applied a ‘reciprocal any overlapping’ method to the CNVRs detected by the two software analyses, and determined the sharing regions by both software programs.

### Gene content and functional analysis

We used the UCSC table browser tool (ISGC ovis aries version 1.0) to identify the gene content located within or partially overlapping with the CNVRs. However, because the sheep genome is not well-annotated compared to the human genome, we used the human orthologs for gene ontology analyses, and DAVID Bioinformatics Resources (http://david.abcc.ncifcrf.gov) was used for further GO functional analysis, including Gene Ontology [13], KEGG pathway [14] and OMIM.

To investigate the gene functions of orthologous human genes in the CNVRs by BLAST search using the UCSC genome browser, we compared these genes in the CNVRs identified by PennCNV with genes that are disease-trait related in the Database of Genomic Variations (DGV) [60]. For this analysis, we used the latest data from the DGV (varation.hg19.v10.nov.2010.txt) downloaded from the DGV website (http://projects.tcag.ca/varation/).

### Validation of CNVR by qPCR

Quantitative PCR experiments (qPCR) were performed using the SYBR green chemistry on the CFX96 Detection System (Bio-Rad, Hercules, CA, USA). The DGAT1 gene was chosen as a reference location for all qPCR experiments [26]. Primer version 5.0 was used to design PCR primers for the selected target CNVs and reference gene. Primer pairs for the control gene fragments and analyzed CNVRs are listed in the Additional file 4: Table S5. qPCR conditions were as follows: The following reagents were used for amplification in 20 μL: 2 μL of DNA, 10 μL of UltraSYBR Mixture (2×), 0.4 μL forward primer (10 μM), 0.4 μL reverse primer (10 μM); thermal cycling was initiated with a 2 min incubation at 50°C followed by a denaturation step from 10 min at 95°C, to 40 cycles of 15 sec at 95°C, and lastly to 1 min at 60°C. All reactions were run in triplicate and included controls without template. The 2^-△△Cq^ method was used to calculate the copy number [61-63], where Cq is the threshold cycle. The CN of the target gene in test sample against reference samples is given by 2 × 2^-ΔΔCq^, where ΔΔCq = [(Cq of target gene in each test sample - Cq of DGAT1 in each test sample) - (average Cq of target gene in reference samples - average Cq of DGAT1 in reference samples)] [23].

### Web resources

UCSC: http://genome.ucsc.edu

PennCNV: http://www.openbioinformatics.org/penncnv/

DAVID Bioinformatics Resources: http://david.abcc.ncifcrf.gov

The Database of Genomic Variations website (DGV): http://projects.tcag.ca/varation/

R program: http://www.R-project.org/

## Competing interests

The authors declare no competing financial interests.

## Authors’ contributions

JL performed all statistical and computational analyses and wrote the manuscript. LZ provided SNP genotype data and coordinated the SNP analysis. LX and JL contributed by giving advice on analyses or by giving advice on the content of the paper. LD and FZ conceived and designed the experiments. XZ, SZ, CW, XZ, participated in phenotype data collection. All authors read and approved the final manuscript.

## Supplementary Material

Additional file 1: Table S1 Size distribution of CNVs identified by PennCNV. **Table S2. **Size distribution of CNVR identified by PennCNV. **Table S3. **Distribution pattern of CNVR across sheep 26 autosome (^a ^The size of chromosome was obtained from NCBI website). Click here for file

Additional file 2: Table S4 Summary of CNV regions and their frequency in sheep genome. Click here for file

Additional file 3: Figure S1 Comparison of CNVRs detected by PennCNV and CNVpartition. Click here for file

Additional file 4: Table S5 Primers information and qPCR results. Click here for file

Additional file 5: Table S6 Gene annotation in sheep CNVRs. Click here for file

Additional file 6: Table S7 CNVRs overlap with known disease-related genes in the DGV database. Click here for file

Additional file 7: Table S8 Comparisons between identified 238CNVRs in this study and the existing other ruminants CNVRs dataset. Click here for file
