# Supplementary material for: Analysis of copy number variations in the sheep genome using 50K SNP BeadChip array
Source: BMC Genomics. 2013 Apr 8;14:229. doi: 10.1186/1471-2164-14-229 (PMC3626776; doi:10.1186/1471-2164-14-229)
Supplement: Additional file 1: Table S1 — Size distribution of CNVs identified by PennCNV. Table S2. Size distribution of CNVR identified by PennCNV. Table S3. Distribution pattern of CNVR across sheep 26 autosome (a The size of chromosome was obtained from NCBI website). [file 1471-2164-14-229-S1.doc]

**Additional file 1:**

**Table S1: Size distribution of CNVs identified by PennCNV**

**Table S2: Size distribution of CNVR identified by PennCNV**

**Table S3: Distribution pattern of CNVR across sheep 26 autosome**

**Table S1: Size distribution of CNVs identified by PennCNV**

| Summary statistics of CNVs (PennCNV output) | Total | Loss | Gain |
| --- | --- | --- | --- |
| Number of CNVs | 3624 | 3416 | 208 |
| Total length(Mb) | 524.1 | 493.6 | 30.5 |
| Average length per CNVs(Kb) | 144.6 |  |  |
| <10Kb |  |  |  |
| ≥10Kb-<50Kb | 561(15.48%) | 540(15.81%) | 21(10.10%) |
| ≥50Kb-<100Kb | 906(25.00%) | 854(25.00%) | 52(25.00%) |
| ≥100Kb-<500Kb | 2102(58.00%) | 1975(57.81%) | 27 (12.98%) |
| ≥500Kb-<1Mb | 52(1.43%) | 46(1.35%) | 6(2.88%) |
| ≥1Mb | 3(0.08%) | 2(0.06%) | 1(0.48%) |

**Table S2: Size distributi**on of CNVR identified by PennCNV

| Summary statistics of CNVR (PennCNV output) | Total | Loss | Gain | Both(gain-loss) |
| --- | --- | --- | --- | --- |
| Number of CNVR | 238 | 219 | 13 | 6 |
| Total length(Mb) | 60.35 | 54.5 | 3.1 | 2.8 |
| Average length per CNVRs(Kb) | 253.6 | 248.7 | 236.4 | 469.9 |
| <10Kb |  |  |  |  |
| ≥10Kb-<50Kb | 5(2.10%) | 5(2.28%) |  |  |
| ≥50Kb-<100Kb | 49(20.59%) | 43(19.63%) | 6(46.15%) |  |
| ≥100Kb-<500Kb | 155(65.12%) | 151(68.94%) |  | 4(66.68%) |
| ≥500Kb-<1Mb | 22(9.24%) | 16(7.30%) | 5(38.46%) | 1(16.66%) |
| ≥1Mb | 7(2.94%) | 4(1.82%) | 2(15.38%) | 1(16.66%) |

**Table S3: Distribution pattern of CNVR across sheep 26 autosome**

| Sheep chromosome | Chromosome size(bp)^a^ | PennCNV | | |  | CNVpartition | | |
| --- | --- | --- | --- | --- | --- | --- | --- | --- |
|  |  | Sum of length of CNVRs | Total number of CNVRs | Proportion(%) of chromosome encompassed by CNVRs |  | Sum of length of CNVRs | Total number of CNVRs | Proportion(%) of chromosome encompassed by CNVRs |
| OAR1 | 299839927 | 6996106 | 28 | 2.33 |  | 722643 | 3 | 0.24 |
| OAR2 | 263237078 | 4433306 | 23 | 1.68 |  | 419666 | 3 | 0.16 |
| OAR3 | 242887918 | 3682899 | 14 | 1.52 |  | 6093104 | 6 | 2.51 |
| OAR4 | 128104666 | 2208989 | 10 | 1.72 |  | 376746 | 2 | 0.29 |
| OAR5 | 117185640 | 4274946 | 10 | 3.65 |  | 2635830 | 3 | 2.25 |
| OAR6 | 129067040 | 2686580 | 11 | 2.08 |  | 12593403 | 1 | 9.76 |
| OAR7 | 108950689 | 2369351 | 10 | 2.17 |  | 1840627 | 5 | 1.69 |
| OAR8 | 97955513 | 3111554 | 15 | 3.18 |  | -- | -- | -- |
| OAR9 | 100831284 | 4153188 | 12 | 4.12 |  | 7904238 | 4 | 7.84 |
| OAR10 | 94216033 | 3107664 | 13 | 3.30 |  | 9114849 | 2 | 9.67 |
| OAR11 | 67137890 | 1642984 | 6 | 2.45 |  | 4107084 | 2 | 6.12 |
| OAR12 | 86457535 | 662944 | 2 | 0.77 |  | 177869 | 1 | 0.21 |
| OAR13 | 89095295 | 596516 | 5 | 0.67 |  | -- | -- | -- |
| OAR14 | 69342551 | 315512 | 1 | 0.46 |  | -- | -- | -- |
| OAR15 | 90133444 | 2158282 | 9 | 2.39 |  | 312583 | 1 | 0.35 |
| OAR16 | 77187635 | 3925649 | 16 | 5.09 |  | 14238834 | 2 | 18.45 |
| OAR17 | 78643424 | 1777705 | 5 | 2.26 |  | 224920 | 1 | 0.29 |
| OAR18 | 72490392 | 755628 | 4 | 1.04 |  | 5462093 | 1 | 7.53 |
| OAR19 | 64988650 | 1501740 | 7 | 2.31 |  | -- | -- | -- |
| OAR20 | 55885198 | 1272025 | 4 | 2.28 |  | -- | -- | -- |
| OAR21 | 55550361 | 2178411 | 8 | 3.92 |  | 169831 | 1 | 0.31 |
| OAR22 | 55886266 | 2047164 | 5 | 3.66 |  | 1140258 | 1 | 2.04 |
| OAR23 | 66770968 | 975590 | 7 | 1.46 |  | 153162 | 1 | 0.23 |
| OAR24 | 45319161 | 513240 | 3 | 1.13 |  | -- | -- | -- |
| OAR25 | 48346598 | 522808 | 4 | 1.08 |  | -- | -- | -- |
| OAR26 | 50101229 | 2479251 | 6 | 4.95 |  | 477170 | 1 | 0.95 |

^a^ The size of chromosome was obtained from NCBI website
