# Supplementary material for: Analysis of copy number variations in the sheep genome using 50K SNP BeadChip array
Source: BMC Genomics. 2013 Apr 8;14:229. doi: 10.1186/1471-2164-14-229 (PMC3626776; doi:10.1186/1471-2164-14-229)
Supplement: Additional file 3: Figure S1 — Comparison of CNVRs detected by PennCNV and CNVpartition. [file 1471-2164-14-229-S3.doc]

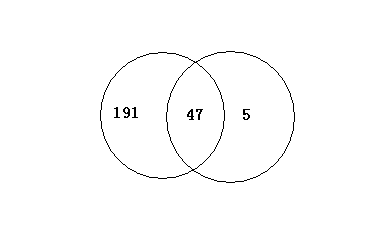
**Additional file 3**

**Figure S1 Comparison of CNVRs detected by PennCNV and CNVpartition.** Left is CNVRs identified by PennCNV output, right is CNVRs identified by CNVpartition output
