# Supplementary material for: Analysis of copy number variations in the sheep genome using 50K SNP BeadChip array
Source: BMC Genomics. 2013 Apr 8;14:229. doi: 10.1186/1471-2164-14-229 (PMC3626776; doi:10.1186/1471-2164-14-229)
Supplement: Additional file 4: Table S5 — Primers information and qPCR results. [file 1471-2164-14-229-S4.doc]

**Additional file 4**

**Table S5**: Primers information and qPCR results

| CNVR ID | Chr | Start | End | Forward and reverse primers | Fragment length(bp) | Frequency  (%) | Type | Number of samples | Confirmed sample | Confirmed | Gene or region |
| --- | --- | --- | --- | --- | --- | --- | --- | --- | --- | --- | --- |
| CNVR16 | 19 | 45775486 | 45897100 | TGTGCGAAACTGAATTTCCTGC | 150 | 2.74 | gain | 7 | 6 | **Yes** | *ARF4* |
|  |  |  |  | GAGATCGGTCCTGAGCCAGC |  |  |  |  |  |  |  |
| CNVR97 | 5 | 51341295 | 51499408 | ACAGGAAAGACAACGGACAAATC | 147 | 3.34 | loss | 8 | -- | **No** | *EGR1* |
|  |  |  |  | GGATCGAAACTGGCACCCT |  |  |  |  |  |  |  |
| CNVR97 | 5 | 51341295 | 51499408 | GCACACCAGGCCGTGATG | 145 | 3.34 | loss | 8 | 1 | **Yes** |  |
|  |  |  |  | CCTTTCCTGCCAGGGGTTC |  |  |  |  |  |  |  |
| CNVR76 | 3 | 166676238 | 167311108 | CCTGACTCCTTGGGACCCG | 142 | 1.50 | loss | 1 | 1 | **Yes** | *AVPR1A* |
|  |  |  |  | GTTGTTGAGTCCCGCAGAGG |  |  |  |  |  |  |  |
| CNVR178 | 16 | 887459 | 1082093 | GCACACCAGGCCGTGATG | 146 | 9.73 | loss | 7 | -- | **No** |  |
|  |  |  |  | CCTTTCCTGCCAGGGGTTC |  |  |  |  |  |  |  |
| CNVR77 | 3 | 180207291 | 180435305 | CTGCCACCTCTGACTTCTGC | 98 | 0.30 | loss | 1 | 1 | **Yes** | *SLC25A3* |
|  |  |  |  | GGAACTGCCTGGGGTCTG |  |  |  |  |  |  |  |
| CNVR87 | 4 | 65357024 | 65666366 | TGTGCGAAACTGAATTTCCTGC | 133 | 6.08 | loss | 7 | -- | **No** |  |
|  |  |  |  | GAGATCGGTCCTGAGCCAGC |  |  |  |  |  |  |  |
| CNVR211 | 21 | 25759271 | 25881397 | ACAGGAAAGACAACGGACAAATC | 128 | 3.04 | loss | 8 | 2 | **Yes** |  |
|  |  |  |  | GGATCGAAACTGGCACCCT |  |  |  |  |  |  |  |
| CNVR214 | 21 | 52788004 | 52907062 | CCTGACTCCTTGGGACCCG | 127 | 6.08 | loss | 6 | -- | **No** |  |
|  |  |  |  | GTTGTTGAGTCCCGCAGAGG |  |  |  |  |  |  |  |
| CNVR69 | 2 | 261198826 | 261718188 | CTGCCACCTCTGACTTCTGC | 136 | 15.20 | loss | 7 | 5 | **Yes** |  |
|  |  |  |  | GGAACTGCCTGGGGTCTG |  |  |  |  |  |  |  |
| Control | 14 |  |  | CCAACCCTGTGCTACGAGC | 131 |  |  |  |  |  | *DGAT1* |
|  |  |  |  | CGGGACCATCCACTGCTGA |  |  |  |  |  |  |  |
| total |  |  |  |  |  |  |  | 60 | 16 |  |  |
